# Quality of Life in Patients With Heart Failure Assisted By Telerehabilitation: A Systematic Review and Meta-Analysis

**DOI:** 10.5195/ijt.2022.6456

**Published:** 2022-06-03

**Authors:** André Luiz Lisboa Cordeiro, Andrêza da Silva Miranda, Halana Melo de Almeida, Paulo Santos

**Affiliations:** 1 Escola Bahiana De Medicina E Saúde Pública, Salvador, Bahia, Brazil; 2 Centro Universitário Nobre, Feira De Santana, Bahia, Brazil; 3 Unidade De Ensino Superior De Feira De Santana, Bahia, Brazil

**Keywords:** Congestive heart failure, Quality of life, Telehealth, Telerehabilitation, Virtual rehabilitation

## Abstract

**Introduction:**

Heart failure (HF) is a syndrome that implies several physical and emotional changes that compromise quality of life. Telerehabilitation is a strategy developed with the aim of involving and motivating cardiac patients to participate in cardiac rehabilitation in their daily routine at home.

**Objective:**

To review the impact of telerehabilitation on the quality of life of patients with HF.

**Methods:**

This is a systematic review using the PICO strategy, with a search conducted in the electronic data sources PubMed, LILACS (Latin American and Caribbean Literature in Health Sciences) and SciELO (Scientific Electronic Library Online), with the following descriptors: heart failure, congestive heart failure, chronic heart failure, distance rehabilitation, virtual rehabilitation, telerehabilitation, telemedicine, quality of life and HRQoL, combined by the Boolean operators “AND” and “OR”, including articles between 2011 and 2021.

**Results:**

Nine articles were found after reading the abstract and titles; five of these met the inclusion criteria. They showed that telerehabilitation contributes to a better quality of life due to the daily increase in mental, social, and sexual activities, exercise tolerance, improvement of symptoms such as edema, fatigue, and dyspnea and reduction of mortality and readmission rates. Telerehabilitation was effective in improving quality of life in patients with HF (mean difference (MD) = -0,22; CI 95% -0.40 to 0.04.

**Conclusion:**

Telerehabilitation was at least as effective as usual care and conventional cardiac rehabilitation in improving the quality of life in patients with HF.

Cardiovascular disease is the chief cause of mortality in humans, with the incidence being primarily due to heart failure (HF) which affects more than 23 million people worldwide. Survival five years after diagnosis is 35%, with an increasing prevalence with age (Rohde et al., 2018).

HF is a syndrome resulting from a structural and/or functional abnormality that causes changes in ventricular filling or ejection, causing reduced cardiac output. HF is associated with risk factors such as obesity, diabetes, high blood pressure, smoking, viral infections, and excessive alcohol consumption (Freitas & Cirino, 2017).

Patients with HF have an impaired quality of life as a result of various physical and emotional symptoms, such as dyspnea and fatigue during exercise, peripheral edema, difficulty sleeping, depression, chest pain, and consequent limitations for performing activities of daily living (Silva & Mendes, 2017).

The analysis of quality of life is considered an important variable in clinical practice and in the production of knowledge in the health area, since it reveals the effectiveness and possible risks of a treatment (Benetti et al., 2010). Quality of life can be measured through the application of questionnaires that reflect the individual's subjective perceptions about their health status in physical, emotional, and social dimensions (Rossi et al., 2011).

Cardiac Rehabilitation (CR) is an intervention that includes physical activity, behavior change, modification of risk factors, and nutritional and psychosocial counseling. CR is recommended for patients with HF because CR can lead to an increase in exercise tolerance, an improvement in functional status, a reduction in dyspnea, better socio-personal and psychological management, and an improvement in quality of life (Tousignant & Mampuya, 2015). In patients with HF, physical training can relieve symptoms, increase exercise tolerance, reduce disability, hospitalization, and mortality; and improve quality of life (Piotrowicz et al., 2016).

Despite evidence on the safety and benefits of CR, there is still a low adherence to CR programs. That is justified by lack of transportation, difficulty in getting around, health problems, age, time, and schedule. CR programs therefore require the implementation of new strategies and interventions to increase their accessibility (Frederix et al., 2015).

Telerehabilitation, defined as the provision of long-distance rehabilitation services through information and communication technologies, is a new strategy with the aim of involving and motivating cardiac patients to participate in CR in their daily home routines (Dinesen et al., 2019). In addition to telerehabilitation, telemonitoring is essential for these patients. Telemonitoring monitors physiological changes, patient diseases, and the evolution of treatments.

Due to the low adherence to CR programs, telerehabilitation is an alternative for patients to benefit from rehabilitation. However, too little is known about the impact of telerehabilitation on the quality of life of patients with HF. The current study therefore aimed to review the impact of telerehabilitation on the quality of life of patients with heart failure.

## METHODS

A systematic review was conducted with the guiding question: “What are the effects of telerehabilitation on quality of life in patients with HF?” The review was completed according to the guidelines of Preferred Reporting Items for Systematic Reviews and Meta-analyzes (PRISMA) (Moher et al., 2009). The review is registered in the PROSPERO (International Prospective Registry of Systematic Reviews) with the number CRD42021233822. The research was structured based on the PICO strategy (Table 1) (Santos et al., 2007).

**Table 1:** PICO Research Strategy

Acronym	Category/Construct	Definition
P	Population	Heart failure patients
I	Intervention	Telerehabilitation
C	Control	Usual care; outpatient rehabilitation, with training on a cycle ergometer; educational session; and, center-based rehabilitation program, education sessions by a multi-professional team and additional home exercises.
O	Outcomes	Quality of life

The following databases were searched: PubMed, LILACS (Latin American and Caribbean Literature in Health Sciences) and SciELO (Scientific Electronic Library Online). The descriptors used for the population were: heart failure, congestive heart failure, and chronic heart failure. Remote intervention, virtual rehabilitation, telerehabilitation, and telemedicine were used for the intervention. Quality of life and HRQoL were used for the outcome. The descriptors were combined with the Boolean operators “AND” and “OR” as per the protocol from the Health Sciences Descriptors (DeCS). The search was conducted between February and March 2021.

### ELIGIBILITY CRITERIA

This study included only randomized controlled trials that addressed the effects of telerehabilitation on the quality of life of patients with HF, were available in English and Portuguese, and were published between 2011 and 2021. For the assessment of quality of life, the following instruments were considered: Medical Outcomes Study Short Form 36 Health Survey Questionnaire; the Minnesota Living with Heart Failure Questionnaire (MLHFQ); and the Euro Quality of Life Instrument-5D (EQ-5D). Exclusion criteria were studies in children, and studies that omitted finalized results, protocols, and evidence of costs.

### DATA EXTRACTION

To extract the selected articles, titles (first stage), abstracts (second stage) and complete reading (third stage) were reviewed to identify studies pertinent to this topic. Then, an exploratory reading of the selected studies was carried out and subsequently, a selective and analytical reading. The data extracted from the articles were summarized with respect to authors, journal, year, title, and conclusions.

The evaluation of the methodological quality of the studies was carried out by two reviewers. When there was disagreement between them, the article was re-read in its entirety and reassessed. If the disagreement persisted, a third reviewer conducted an assessment and generated a final decision.

### METHODOLOGICAL QUALITY ASSESSMENT

Methodological quality was assessed according to the PEDro12 scale criteria, which scores 11 items, namely: 1- Eligibility criteria, 2 - Random allocation, 3 - Hidden allocation, 4 - Baseline comparison, 5 - Blinded individuals, 6 - Blinded therapists, 7 - Blinded evaluators, 8 - Adequate follow-up, 9 – Intention-to-treat analysis, 10 - Comparisons between groups, 11 - Point estimates and variability. Items are scored as present (1) or absent (0), generating a maximum possible sum of 10 points, with the first item not counting (Maher et al., 2003).

Whenever possible, PEDro scores were extracted from the PEDro database itself. When the articles were not found in the PEDro database, two trained reviewers evaluated the article using the PEDro scale. Studies were considered to be of high quality if they had a score equal to or greater than six. Studies with a score of less than six were considered to be of low quality.

### SYNTHESIS OF RESULTS

The presence of heterogeneity was evaluated using the Chi2 test and the I2 statistic. The latter statistic illustrates the percentage of variability in effect estimates from heterogeneity rather than sampling error.

### STATISTICAL ASSESSMENT

The mean difference between groups and the respective 95% confidence intervals were calculated and used to quantify the effect of continuous outcomes. For the meta-analyses in which the studies used the same scales, the results were presented as mean difference (MD) and 95% confidence intervals. Otherwise, the effects were calculated using standardized mean difference (SMD) and 95% confidence intervals. The effect size of the interventions was defined as small (MD < 10% of the scale or SMD < 0.4); moderate (MD = 10% to 20% of the scale or SMD = 0.41 to 0.7) or large (MD > 20% of the scale or SMD > 0.7).

## RESULTS

**Figure 1:**
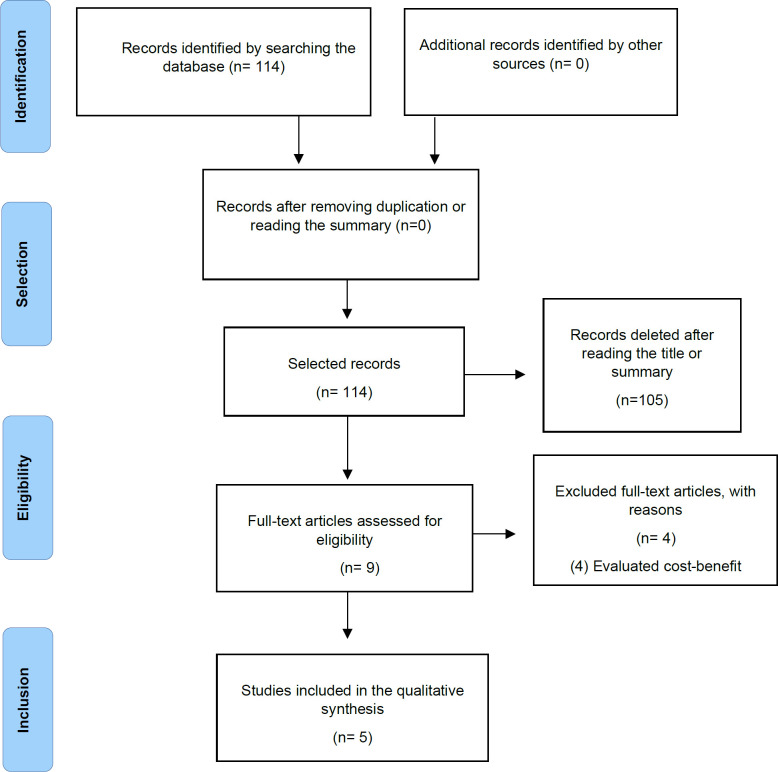
PRSIMA Flowchart

*Note*. The PRISMA flowchart depicts the process for obtaining randomized clinical trials based on telerehabilitation as an intervention for the quality of life of patients with heart failure.

Nine articles were found after reading the abstract and titles, of which only five were selected according to the inclusion criteria. Articles with a cost-benefit study (4) were removed. The PRISMA flowchart in Figure 1 shows all the criteria used for the selection of articles.

The methodological quality assessed by the PEDro scale is shown in Table 2, including articles with scores between six and eight.

**Table 2:** Methodological Evaluation of the Studies Included in This Review, Using the PEDro Database Scale

		Bernocchi et al., 2018	Hwang et al., 2017	Peng et al., 2018	Piotrowicz et al., 2015a	Piotrowicz et al., 2015b
01	The eligibility criteria have been specified.					
02	Subjects were randomly assigned to groups.	✓	✓	✓	✓	✓
03	The allocation of the subjects was secret.	✓	✓	✓		
04	Initially, the groups were similar with regard to the most important prognostic indicators.	✓	✓	✓	✓	✓
05	All subjects were blinded					
06	All therapists who administered the therapy were blinded				✓	
07	All evaluators who measured at least one key result were blinded.	✓	✓	✓		
08	Measurements of at least one key result were obtained in more than 85% of the subjects initially distributed among the groups.			✓	✓	✓
09	All subjects from whom measurements of results were presented received the treatment or the control condition according to the allocation, or when this was not the case, the data was analyzed for at least one of the key results by “intention to treat”.	✓	✓	✓	✓	✓
10	The results of intergroup statistical comparisons have been described by at least one key result.	✓	✓	✓	✓	✓
11	The study presents both precision measures and measures of variability for at least one key result.	✓	✓	✓	✓	✓
**Sum of Scores**		**7/10**	**7/10**	**8/10**	**7/10**	**6/10**

The five studies included in this systematic review discuss the quality of life of patients with HF assisted by telerehabilitation, published from 2015 to 2018. A summary of the methods used and results achieved are shown in Table 3.

**Table 3:** General Data from Included Randomized Controlled Trials on the Quality of Life of Patients with Heart Failure Assisted by Telerehabilitation

Author/Year	Sample	Average age	Objective	Intervention	Intervention protocol	QOL assessment	Results
Bernocchi et al., (2018)	112	70 years	To investigate the feasibility and effectiveness of a home telerehabilitation program in patients with combined COPD and CHF.	Intervention group: Educational session and personalized exercise program for each patient, until dyspnea and/or muscle fatigue is achieved. Control group: Received the standard care program including medications and oxygen prescription, visits from the general practitioner, and in-hospital check-ups on demand. At enrolment in the study, patients were instructed in an educational session about the desirability of maintaining a healthy lifestyle and were invited to practice daily physical activity as preferred.	Basic level: 15-25 minutes of exercise with a mini-ergometer with no load and 30 minutes of callisthenic exercises, three times a week and free walking twice a week. High level: 30 to 45 minutes of muscle strengthening exercises with weights and walking on a pedometer, three to seven days a week.	MLHFQ	The quality of life was higher in the group that carried out the telerehabilitation, with p = 0.0001.
Hwang et al., (2017)	53	67 years	Determine the effectiveness and safety of a heart failure rehabilitation program delivered to each participant's home through an online telerehabilitation system.	Intervention group: Home telerehabilitation program and education sessions by a multiprofessional team. Control group: Center-based rehabilitation program, education sessions by a multiprofessional team and additional home exercises.	Both programs take place over 12 weeks, with two sessions per week. 10 minutes of warm-up, 40 minutes of aerobic exercise and 10-minute strength and relaxation.	MLHFQ and EQ-5D	No differences were found between groups in quality of life.
Peng et al., (2018)	98	66.3 years	Examine the effects of the telehealth exercise training program on health outcomes in patients with HF in China.	Experimental group: telehealth exercise training program, for two months. Control group: The patients in the control group in the usual care setting were not given any type of instruction regarding exercise	First stage: resistance exercises, with three sessions of 20 minutes per week. Second stage: resistance exercises and muscle strengthening, with five sessions of 30 minutes per week.	MLHFQ	The quality of life was higher in the group that carried out the telerehabilitation, with p = 0.05.
Piotrowicz et al., (2015a)	131	56.4 ± 10.9 years	To assess changes in quality of life in patients with HF after home cardiac rehabilitation by telemonitoring versus standard outpatient cardiac rehabilitation.	Experimental group: telemonitored home rehabilitation, based on walking training. Control group: participate in meetings with a psychologist, held three times a week, on the same day as training sessions.	Warm-up lasting 5 to 10 minutes, basic aerobic resistance training for 10 to 30 minutes and 5 minutes of cooling down, three times a week for eight weeks.	SF-36	There were no statistically significant differences between the groups in relation to the total QOL index.
Piotrowicz et al., (2015b)	111	54.4 to 62.1 years	To evaluate the safety, efficacy, adherence and acceptance of home telemonitored Nordic walking training in patients with HF, including those with implantable cardiovascular electronic devices.	Training group: Walking training, with a telemonitoring device. Control group: Patients in the CG receiving ‘usual care’ were not provided with a formal exercise training prescription and did not perform supervised rehabilitation. All patients, regardless of the treatment group, received recommendations for suitable lifestyle changes and self-management according to ESC guidelines.	Duration of eight weeks, five times a week; 5-10 minutes of warm-up, 15-45 minutes of training and 5 minutes of cool-down.	SF-36	The quality of life was higher in the group that carried out the telerehabilitation, with p = 0.001.

*Note.* COPD: Chronic Obstructive Pulmonary Disease; EQ-5D: Euro Quality of Life Instrument-5D; HF: Heart Failure; CHF: Chronic Heart Failure; MLHFQ: Minnesota Living with Heart Failure Questionnaire; QOL: Quality of Life; SF-36: Medical Outcomes Study Short Form 36 Health Survey Questionnaire.

The total sample of articles included 505 participants of both sexes. There was a predominance of males; mean participant age was over 50 years; comorbidities included diabetes mellitus and hyperlipidemia; the etiology of HF was mainly due to ischemic heart disease and myocardial infarction; and the functional level of participants per the classification of the New York Heart Association (NYHA) varied from II to III.

Piotrowicz et al. (2015a) demonstrated an improvement in exercise capacity and an increase in mental, social, and sexual activities of daily life, factors that contributed to a significant improvement in quality of life. Research by Peng et al. (2018) also showed improvements in quality of life, though related that to factors such as improvement in edema, fatigue, shortness of breath and increased exercise capacity. Other articles (Bernocchi et al., 2018; Hwang et al., 2017; Piotrowicz et al., 2015b) reported that telerehabilitation is capable of promoting a significant improvement in quality of life.

For comparison with the intervention, Piotrowicz et al. (2015b) considered habitual care, which included recommendations for changes in lifestyle and self-care, whereas Peng et al. (2018) also considered usual care, but limited it to information at discharge and regular follow-up visits to the clinic. In the other studies, the educational session was applied, with the objective of maintaining a healthy lifestyle and practicing some preferred daily physical activity, or rehabilitation in a center and home exercises.

### QUALITY OF LIFE

Five studies analyzed the impact of the telerehabilitation on the quality of life. For the meta-analysis of this comparison, a random model was used (I^2^ = 68%, df = 4, p = 0.01) in which there was a statistically significant difference between the groups with respect to the comparison between the telerehabilitation and the control (difference between means = -0.22; 95% CI -0.40 to 0.04).

## DISCUSSION

Based on our review of pertinent studies, telerehabilitation can improve the quality of life of patients with HF, and it is associated with factors such as increased exercise capacity, increased mental, social and sexual function and improvement of symptoms such as edema, fatigue and dyspnea.

Piotrowicz et al. (2015a) found that patients who participated in a telerehabilitation program felt safe even though they were rehabilitated at home, due to the modes of monitoring and supervision. They observed an improvement in the ability to perform exercise and activities of daily living, which contributes to improved quality of life. It is known that quality of life is associated with aspects beyond physical capacity, in which, with the practice of exercises, it is also possible to contribute to good mental and social conditions.

Exercise programs are essential for patients with HF to improve physical function and quality of life. Hwang et al. (2017), when comparing the center-based rehabilitation program with the telerehabilitation program, including a similar exercise protocol, showed that telerehabilitation was not inferior, as there was no difference between the two groups of intervention with respect to the quality of life index.

Also comparing the programs, although with different training modalities, Piotrowicz et al. (2015b) concluded that telemonitored home cardiac rehabilitation provided an improvement similar to outpatient rehabilitation in terms of total quality of life index. These results are consistent with the concept that regular physical training, regardless of the exercise mode, can contribute to an improvement in psychological state and to greater independence in the performance of activities of daily living.

Physical exercise is considered fundamental for the treatment of HF, as it can improve tolerance for physical work, reduce symptoms related to the syndrome, and generate physiological as well as psychological changes that are associated with the control of risk factors and with the improvement of quality of life (Brum et al., 2011).

Improvements in quality of life may also be related to an improvement in symptoms such as edema, fatigue, and shortness of breath, and to an increase in exercise capacity, as shown in the study by Peng et al. (2018). In addition, consultations and monitoring with the multidisciplinary team are also helpful, as they offer greater support in performing exercises as well as emotional support.

Clinical manifestations such as edema, dyspnea, and decreased tolerance to activities of daily living, also influence the social, psychological, and spiritual aspects of patients with HF, as these interfere with the quality of life and well-being in a systemic way (Paz et al., 2019).

The value of telemonitoring derives from daily contact with the monitoring center. Patients repeatedly receive information about the importance of the intervention, which can improve compliance with protocols and advice.

Bernocchi et al. (2018) stated that patients often have CHF and COPD concomitantly due to common risk factors, which makes them more fragile and limited than if they just had one of these conditions in isolation. They reported that individuals in the intervention group experienced a reduction in dyspnea and disability, improvements in quality of life, and fewer hospital readmissions. This indicates that telerehabilitation can be feasible, safe, and effective for individuals with both conditions.

Patients with chronic conditions need to be encouraged to follow rehabilitation programs, since physical training increases the maximum oxygen volume and resistance and improves endothelial and myocardial function (McMahon et al., 2017).

Telerehabilitation is a relatively new approach to care delivery with the potential to safely and effectively monitor clinical symptoms during rehabilitation exercises through real-time communication with health professionals, thereby reducing the risk of mortality and the number of hospitalizations related to heart failure and improving quality of life (Hwang et al., 2017).

The present study has limitations. There was heterogeneity in the study protocols; the number of studies eligible for analysis was small; and the total number of participants was small.

## CONCLUSION

Based on the results, it is concluded that telerehabilitation can contribute to improvement in the quality of life of patients with HF as effectively as other commonly available interventions. Possible mechanisms for this effectiveness include improvements in factors such as increased exercise capacity, independence in carrying out activities of daily living, reduction of symptoms, and increased mental, social, and sexual function. Further, telerehabilitation might be as effective as conventional outpatient cardiac rehabilitation in reducing the risk of mortality and the number of hospitalizations in individuals affected by this health condition.
